# Problematic situations related to social media use and competencies to prevent them: results of a Delphi study

**DOI:** 10.1038/s41598-024-55578-5

**Published:** 2024-03-04

**Authors:** H. Lahti, M. Kulmala, N. Lyyra, V. Mietola, L. Paakkari

**Affiliations:** Faculty of Sport and Health Sciences, University of Jyväskylä, P.O. Box 35 (L), 40014 Jyväskylä, Finland

**Keywords:** Social media, Problematic situation, Competency, Health, Wellbeing, Adolescent, Delphi, Psychology, Human behaviour, Risk factors, Health policy, Public health

## Abstract

A three-round Delphi method was used to study the problematic situations that adolescents may encounter when using the social media, and the competencies needed to address these situations. A panel of Finnish experts (*N* = 22) provided an open-ended list of problematic situations and competencies in 2020–2021. These were then evaluated and ranked according to their significance. The experts provided an information-rich list of both problematic situations and competencies. Finally, 16 problematic situations and 19 competencies were ranked in order of importance by the experts. The most important problematic situations were direct and indirect cyberbullying and sexual harassment. The most important competencies were the ability to act responsibly, knowing what kinds of activity are prohibited, and knowing whom to contact on exposure to cyberbullying or harassment. The findings can be used in developing policies, recommendations, and solutions aimed at counteracting the harmful effects of social media on wellbeing during adolescence.

## Introduction

**S**ocial media spaces encompass social networking sites such as Facebook and Instagram, and instant messaging applications such as WhatsApp and Snapchat. These can serve as important growth and developmental contexts for adolescents^1^. Furthermore, because the online world overlaps with the offline world, the online world may help adolescents to “navigate important developmental issues from their offline lives,” including sexuality, identity, and health^2^. The social media provide a venue for connection, for identity expression and formulation, and for comparison with others and the establishment of social norms^3^. Among adolescents, social media use constitutes a prime activity for entertainment, information-seeking, and communication^4^, with an international European Commission report indicating that young people between the ages of 10 and 18 spend up to 7.5 h online per day^5^. Furthermore, the EU Kids Online 2020 report indicates that 69% of persons aged 12–14 and 81% of persons aged 15–16 go online *several times each day* or *all the time*^6^. Consequently, ensuring a safe and secure social media environment for adolescents has been incorporated as a key component in the European strategy for a Better Internet for Kids (BIK+)^7^ and in the EU Strategy on the Rights of the Child^8^.

Although the social media contribute positively to adolescents’ lives^3,9^, adolescents are vulnerable to various problematic situations while navigating and experimenting with the social media^1,9,10^. This may be due to their susceptibility to peer pressure, and to having limited self-regulation skills, as well as other competencies that would prevent or deal with such situations^1,11,12^. In this paper, problematic social media situations encompass risky or threatening situations which may cause negative effects on adolescents’ health and wellbeing. The competencies in question combine skills, knowledge, and awareness sufficient to prevent problematic situations arising from social media and to deal with problematic situations if they arise^13^.

Previous studies have identified a range of problematic situations related to adolescents’ social media use; these may be grouped as those with (1) *direct* or (2) *indirect* consequences on their health and wellbeing^2,6^. The situations with *direct* health consequences involve situations such as cyberbullying^14^ and sexual harassment^15^, both of which have been associated with lower life satisfaction^16^, and psychosomatic problems such as depressive symptomatology^17,18^. Cyberbullying is also associated with a greater likelihood of self-harm and suicide^19,20^. According to EU Kids Online 2020, 14% of adolescents report being a victim, while 8% report having been a bully at least a few times. Furthermore, 23% report having been a victim of aggressive behavior, and 14% report having been aggressors themselves. As regards sexual messages, 22% mention having received sexual messages over the past year, with 17% having received unwanted sexual requests at least a few times, and 6% having sent sexual messages^6^. The convergence of different forms of bullying and sexual harassment increases the likelihood of even more negative health impacts, as compared to experiencing just one type of victimization^16^. There is the further possibility of being in contact with strangers over the social media, which may increase the likelihood of being subjected to sexual harassment^18^.

The situations with *indirect* health and wellbeing consequences could include the exposure to and sharing of a variety of harmful and provocative materials^1,6^. These can include risky social media challenges^21^, as well as images and other content associated with high-risk behavior. There is evidence that social media content discussing risk behaviors (such as substance misuse) can potentially support beneficial attitudes regarding these behaviors^22^. Furthermore, social media challenges and risky selfie behavior^23^ may encourage the performance of dangerous acts (e.g., climbing a cliff or onto a train) by which adolescents may seek to foster their social identity. Risk-taking behavior may result from a general desire to satisfy risk-taking needs, but also from a desire to connect with deviant peers and communities, and to seek peer approval^1^. Adolescents’ desire for peer approval may be strengthened by the neural sensitivity of the socio-emotional system, which enhances the anticipated reward value of risk behaviors that are likely to be seen by peers^24^.

Harmful and provocative material includes the plethora of appearance-focused content on popular adolescent social media platforms^6,25^. Exposure to such content may lead to distorted images of reality, and these may in turn lead to objectifying self-concepts, impossible body standards, and lowered self-esteem, especially among girls^10,26^. Furthermore, the EU Kids Online 2020 report^6^ indicates that harmful and provocative content includes false information, racial discrimination, and hate speech. These can easily spread through adolescent online social networks and cause anxiety and distress^27,28^, contributing to victimization, polarization, discredited stereotyping, and deterioration of trust towards authorities^29^. In Europe, 8–17% of adolescents aged 12–16 report that they have faced harmful content online at least monthly^6^. The same report notes that “exposure to different types of harmful content is interrelated—i.e., if a child sees one type of content, it is more likely that the same child will also see other types of harmful content.” Approximately 10% of adolescents mentioned having encountered content on how to commit suicide, how to physically harm or hurt oneself, experiences of taking drugs, and ways to be thinner. In addition, 17% had encountered hate messages attacking particular groups or individuals^6^.

Through social media, adolescents are also confronted with advertising that could have harmful effects on their health and behavior^30^. The social media allow marketers to adapt their messages to reach millions of adolescents, via targeted ads based on content that adolescents have previously viewed or posted on their profiles^31^. Furthermore, the social media have broadened the types of products adolescents are now exposed to. For example, major alcohol brands maintain a strong presence in popular adolescent social media platforms such as TikTok^32^, and could thus impact on adolescents’ health through endorsing alcohol consumption.

Other possible indirect effects on adolescents’ health and wellbeing derive from privacy issues. The content that adolescents choose to share on any social media platform becomes to some degree public, and removal of such content can be difficult or impossible^33^. Furthermore, research has demonstrated that adolescent privacy practices vary significantly, and that even adolescents who understand how to manage privacy settings may choose not to do so^6,33^

The problematic situations related to adolescent social media use are diverse, as are the competencies for preventing and resolving such situations. Previous studies have identified an array of social media-related competencies, which include the self-regulatory skills needed to limit one’s time on the social media and develop healthy usage patterns^34^, together with the skills to maintain one’s privacy^33^, protect oneself from inappropriate material^34^, and limit one’s online disclosure of information^35^. Attention has been drawn to adolescents’ need for cooperative conflict-resolution, ethical skills, and empathy^36,37^. There has also been an emphasis on general media literacy skills (e.g., to protect oneself from mis- and disinformation)^37^, and health literacy^38^, plus the abilities to talk about problematic social media situations with trusted adults, and to seek help if needed^39^. Many of the competencies mentioned above could help in addressing problematic situations in the social media from the perspective of both perpetrators and victims. For example, conflict-resolution skills and empathy have been shown effective against cyberbullying^36^.

Problematic situations can have long-term negative effects on adolescents’ development, wellbeing, and health. However, relatively little research has been conducted on problematic situations related to adolescents’ social media use and their competencies in addressing these situations. One general conclusion from studies on problematic situations relating to the social media (regarding e.g., school interventions) has been that there is a need for a (possibly gradually achieved) expert consensus on (1) the most important problematic situations bound up with adolescents’ social media use, and (2) the kinds of competencies that will be most effective in preventing such situations^34,37^. Such research is essential if one is to develop policies, recommendations, and solutions that could target and prevent the harmful effects of the social media on the wellbeing of adolescents^10^. With this aim in view, the research questions for the present study were:

- RQ1. What are the most important problematic situations that adolescents may encounter when they use the social media?
- RQ2. What kinds of competencies do adolescents need to prevent problematic situations arising from the social media, and to deal with problematic situations if they arise?

## Material and methods

The Delphi method was utilized for this study. The Delphi method combines quantitative and qualitative processes that draw anonymously on selected experts’ opinions, and it aims to obtain a group consensus on a phenomenon^40,41^. The Delphi method has been deemed appropriate where scientific knowledge on the topic studied is scarce^40^, and it has been seen as useful when qualitative methods (such as face-to-face data collection) are impractical^41^. In this study, a three-round survey process was employed over a period of seven weeks^42^. Before conducting the Delphi study, the research team developed the questions internally and conducted pilot testing with selected experts. The aim of the pilot testing was to ensure the usability and comprehensibility of the questions for external participants. This was done by ensuring that the questions in each round were suitable and understandable, and thus appropriate for the study purposes^43^. Pilot testing was carried out prior to each round of the Delphi study.

### Participants and procedure

In previous Delphi studies, the sample sizes have varied from three to several hundred^44^; however, the majority of Delphi panels consist of under fifty experts^45^. In the present study, previous Delphi studies on health promotion were used as a guideline for selecting an appropriate number of participants, in conjunction with the guidelines provided by Okoli and Pawlowski^46^. Thus, the present study was based on the subjective opinions of *22 pre-selected experts*.

Because a Delphi study is a group-decision mechanism requiring experts with a deep understanding of the issues^46^, the present study employed purposeful sampling to identify “information-rich participants.” Specifically, we employed maximum variation sampling^47^ to gather diverse expert viewpoints on the phenomenon in question and thus gain a meaningful consensus on the topic^45^. The sample recruitment process was based on the Knowledge Resource Nomination Worksheet (KRNW)^46^. Following KRNW—which is designed to help categorize experts before identifying them—the relevant knowledge areas, skills, practitioners, academics, and organizations were initially identified. The worksheet was then populated with the names of relevant individuals, pinpointed either through organizational websites or expert publications. Sub-lists were created for each area of expertise, and experts were ranked and categorized appropriately, leading to the formation of a panel structure. The experts were selected in order of their ranking, profession, geographical position, and area of expertise to achieve a versatile panel that could provide multiple viewpoints on the subject matter. Complementary expertise was pursued by selecting many different kinds of specialists, hence, not merely (for example) researchers. The chosen experts were then invited, with a request to propose an alternative participant in case they were unable to participate.

On the basis of the KRNW, the experts chosen for this study were researchers from the fields of media education, educational science, psychology, health education, and information research, with inclusion also of teachers and principals working in primary and secondary education and in high schools. There were also other proven experts from the fields of the media, plus professionals in the social and healthcare fields, such as psychologists, child psychiatrists, medical doctors, and youth workers. Data collection was implemented via an electronic questionnaire sent to the selected participants by email. Anonymity is a key component of a Delphi study, with the aim of freely facilitating views on the topic; thus, the email was sent to the selected persons with no possibility to trace an answer to a particular individual. The participants did not know the content of other responses, nor the personality of other respondents. The collation of the responses was undertaken by the research group. The Delphi was performed in Finnish, which was the native language of the experts.

### The first round of the Delphi study

The goal in the first round was to encourage experts to freely produce ideas on the research phenomenon, and to generate questionnaire items for the second round^48^. The first round consisted of two open-ended questionnaires in which experts were asked to list (1) problematic situations that adolescents may encounter when they use the social media, and (2) competencies that adolescents need to prevent and deal with problematic situations in the social media. Five members of the research team carefully considered the answers that the experts in the first round had provided. The qualitatively differing problematic situations and competencies were identified and listed (separately), and the overlaps from the responses were removed. While reading the expert responses, the members of the research team acted as critical friends for each other. This approach can be described as a critical dialogue between researchers, in which their understandings are shared and mutual critical feedback is given^49^. The various viewpoints of the team members were thus positioned as resources for challenging and expanding the interpretations^49^. The responses were (re)formulated as statements for the second round, ensuring loyalty to the original responses. Thus, all qualitatively differing problematic situations and competencies were identified and listed.

### The second round

For the second round, an online questionnaire was created containing a collection of items mentioned by participants. The problematic situations and competencies were listed separately. Within each list, the items were presented in random order. In building the items, the wording used by participants was followed as closely as possible^48^. The experts were asked to rate the importance of each item on a 7-point Likert scale using the questionnaire. The scale for the problematic situations and the scale for the competencies both ranged from 1 = *not at all important* to 7 = *very important*. The experts’ responses were quantitatively analyzed based on previous Delphi literature^48,50^. To determine the most important items, the modes, medians, and means were computed. In addition, standard deviations and Z-scores (standardized scores with sample mean = 0, standard deviation = 1) were calculated. Agreement percentages were also inspected. Firstly, calculation was made of the number of agreeing pairs of respondents divided by the number of all possible pairs of respondents in the dataset. Additionally, the proportion of respondents who rated an item as among the top x most important items (abbreviated henceforth as agree % ≥ x) was determined for different values of x. The most important items were listed and utilized to create a new questionnaire for the final round (i.e., round 3).

### The third round

In the third round, the experts were asked to select and rank the eight most important problematic situations and competencies separately. Items that did not make the top-eight list were given a value of 0. The ranking was applied to the items that emerged as the most important in the second round, based on the quantitative analysis. The sum scores, the mean, and the agreement percentages of the experts’ responses were analyzed to determine the most important problematic situations and competencies according to the experts’ opinion.

### Ethical approval

The Ethical Committee of the University of Jyväskylä was consulted and concluded that applying for ethical approval was not necessary due to the use of anonymous procedures. All three rounds of the Delphi study contained questions regarding willingness to participate. At this point, the participants approved the privacy notice compliant with the European Union's General Data Protection Regulation (GDPR)^51^. All research procedures followed the responsible conduct of research guidelines and regulations of the Finnish National Board on Research Integrity (TENK)^52^. Informed consent was obtained from all participants.

## Results

### The first round

The panel for the first round consisted of 19 experts. The two open-ended questionnaires gave a list of 125 problematic situations that adolescents may encounter related to social media use, and 82 competencies required to address problematic situations. After careful consideration of the qualitative similarities in the content, 29 problematic situations (Table 1) and 24 competencies (Table 2) were formulated.

**Table 1:** Problematic situations that adolescents may encounter when they use social media, as identified by an expert panel.

Problematic situation	Median	Mean	Mode	Std	Agreement % ≥ 5	Agreement % ≥ 6	Agreement % = 7	Z-Score
**Exposure to direct cyberbullying (vicious behavior, anonymous bullying, public humiliation, name-calling)**	7	6.50	7	0.91	95	95	64	1.98
**Exposure to indirect cyberbullying (becoming excluded from digital communities, online gossip)**	6	6.14	7	0.89	95	77	41	1.26
**Incapacity to manage time spent on social media**	6	6.00	7	1.20	82	68	50	0.99
**Lack of knowledge and skills to critically address social media content**	6	6.00	7	1.11	91	73	41	0.99
**Exposure to pressures regarding appearance; an appearance-oriented world view**	6	6.00	7	0.98	95	64	41	0.99
**Excessive time spent on social media, and increased screen time**	6	5.95	7	1.33	86	73	45	0.90
**Exposure to racism**	6	5.91	7	1.11	86	64	41	0.80
**Reduced quality/quantity of sleep through the use of social media**	6	5.86	7	1.36	86	64	45	0.71
**Addiction to social media use (i.e., compulsive and uncontrolled use)**	6	5.86	7	1.17	91	59	41	0.71
**The need to be constantly available in order not to be excluded (fear of missing out)**	6	5.59	7	1.30	82	55	32	0.17
**Exposure to online scams**	6	5.36	7	1.65	68	55	36	− 0.28
**Exposure to sexual harassment and molestation**	6	5.86	6	0.99	86	73	27	0.71
**The child or adolescent behaves offensively on social media and does not understand the emotional content of messages (low emotional skills)**	6	5.82	6	1.05	82	73	27	0.62
**Sharing without permission the private and sensitive information or files of other people**	6	5.82	6	0.91	91	68	23	0.62
**Sharing of one’s own personal, private, and sensitive information or files**	6	5.62	6	1.02	90	57	19	0.23
**Exposure to negative behavior/provocative material shared by others (e.g., images or video footage of violence, at-risk situations, intoxicants, or gambling)**	6	5.10	6	1.55	67	52	14	− 0.81
Social media having an unfavorable effect on concentration and attention when studying	6	5.82	5	0.96	95	55	32	0.62
Exposure to a distorted image of reality	6	5.68	5	1.17	86	55	32	0.35
Exposure to social media challenges that are harmful or dangerous to health	5	4.95	6	1.25	64	36	9	− 1.09
Valuing others on the basis of social media profiles (e.g., number of followers or likes)	5	5.36	5	1.29	77	45	23	− 0.28
Inadvertent or intentional dissemination of false information (e.g., fake news and conspiracy theories)	5	5.27	5	1.28	77	36	23	− 0.46
Exposure to distorted or false information (e.g., fake news, conspiracy theories)	5	5.10	5	1.30	71	38	14	− 0.81
Seeking out material that could provoke negative behavior by the person encountering it (e.g., porn sites or violent sites; joining groups that encourage risky behavior)	5	5.09	5	1.54	77	41	18	− 0.82
Exposure to poor role models, and their glorification	5	5.05	5	1.50	73	36	18	− 0.91
Exposure to commercial marketing (tempting to buy something that a young person cannot afford, such as in-game purchases)	5	5.00	5	1.35	77	32	14	− 1.00
Exposure to targeted influence (e.g., the social media front page is modified according to the person’s previously searched material)	5	4.82	5	1.44	68	32	9	− 1.36
Exposure to an excessive information flood	5	4.64	5	1.50	64	32	5	− 1.72
Poorly protected social media profiles	5	4.57	5	1.54	57	29	10	− 1.85
Exposure to identity theft	5	4.86	4	1.42	55	36	14	− 1.27

**Table 2:** Competencies required to prevent/deal with problematic social media situations, as identified by an expert panel.

Competency	Median	Mean	Mode	Std	Agreement % ≥ 5	Agreement % ≥ 6	Agreement % = 7	Z-Score
**Knowing whom to contact when exposed to cyberbullying, harassment, or sexual harassment**	7	6.73	7	0.55	100	95	77	1.33
**Ability to act responsibly and without offending others on social media**	7	6.59	7	0.80	95	91	73	1.04
**Ability to act empathetically and with respect for others on social media**	7	6.59	7	0.80	95	91	73	1.04
**Ability to assess the trustworthiness of a previously unknown online friend**	7	6.55	7	0.74	95	95	64	0.94
**Knowing what kinds of activity are prohibited (identity theft, sexual harassment, dissemination of information, defamation)**	7	6.55	7	0.91	91	91	73	0.94
**Ability to ask for help from a trusted adult if necessary**	7	6.50	7	0.74	100	86	64	0.84
**Ability to assess what contents are suitable for publication or sharing**	7	6.50	7	0.80	95	91	64	0.84
**Ability to protect personal privacy (e.g., passwords and profile privacy settings)**	7	6.50	7	0.74	100	86	64	0.84
**Ability to manage time spent on social media**	7	6.41	7	0.96	95	91	59	0.65
**Ability to assess the trustworthiness of published information**	7	6.41	7	0.73	100	86	55	0.65
**Knowing where to report inappropriate material**	7	6.36	7	0.85	95	86	55	0.55
**Ability to identify, process, express, and regulate emotions on social media**	6	6.32	7	0.78	100	82	50	0.46
**Ability to compare information published in different data sources**	6	6.14	7	0.83	100	73	41	0.07
**Having knowledge and skills on how to apply security practices to protect privacy (one’s own and that of others)**	6	5.86	7	1.25	86	59	45	− 0.51
**Knowing one’s own rights (e.g., right to information, privacy, and freedom of expression)**	6	6.09	6	0.87	95	77	36	− 0.03
**Ability to explain how social media affect one’s self-image and self-esteem**	6	5.95	6	1.13	95	77	32	− 0.32
**Ability to evaluate the credibility of social media posts; knowing that information given on social media is not the whole truth about the publisher's life**	6	5.95	6	0.90	95	68	32	− 0.32
**Ability to identify problematic social media situations in one’s daily life**	6	5.73	6	1.16	91	68	23	− 0.81
**Ability to assess one's own behavior and that of others on social media**	6	5.73	6	0.83	91	68	14	− 0.81
Ability to explain how social media use can affect one’s health	6	5.55	6	1.10	82	59	18	− 1.19
Ability to assess the distribution and persistence of one’s own publications (digital footprint)	6	5.77	5	0.97	91	59	27	− 0.71
Knowing matters related to the privacy, publicity, and ownership of apps and sites	5	5.27	6	1.35	77	50	18	− 1.78
Ability to give examples of possible social media problems	5	5.24	5	0.94	81	29	14	− 1.85
Ability to explain the means of influencing used by commercial operators on social media (marketing, influencing)	5	5.23	5	0.97	77	32	14	− 1.87

### The second round

Twenty-two experts participated in the second round. The problematic situations and the skills were listed separately from each other, each in random order, to avoid influencing the results. Based on the quantitative analyses, the experts considered most of the 29 problematic situations and 24 competencies to be important (i.e., having medians ≥ 5, modes ≥ 5, with one exception; Tables 1, 2). For the subsequent (third) round, the cut-off criteria were set at a median and mode of ≥ 6, a mean of ≥ 5, and a Z-score ≥  − 1. The decision was based on the need to have a sufficient number of high-importance items for further evaluation and selection in the third round, but also to gradually move towards identifying the most important problematic situations and competencies among adolescents (i.e., to narrow down the responses)^43^. According to statistical assessment, a more lenient cut-off would have yielded too many items, whereas a more stringent cut-off would have overly constrained the pool of items. Nevertheless, we have listed all the items and their corresponding values in Tables 1 and 2, recognizing that no generally accepted cut-off criteria exist in the literature^53^. The selected cut-off yielded 16 problematic situations and 19 skills (indicated by bold text in Tables 1, 2).

### The third round

In the final round, 17 experts participated in the questionnaire. In this round, the experts were asked to identify and then rank the eight most important problematic situations and competencies among the 16 problematic situations and 19 competencies that remained from the second round. The most important item received eight points from the participants and the eighth most important received one point, yielding a theoretical maximum of 136 if all of the participants had chosen the same item as the most important. The findings (Tables 3, 4) indicate that the responses varied across the items, but that all of the items were mentioned in the lists of the eight most important items provided by the respondents overall.

**Table 3:** Problematic situations that adolescents may encounter when they use social media; ranked in order of importance by an expert panel.

Problematic situations	Sum	Mean	Agreement % = 8	Agreement % ≥ 4	Agreement % ≥ 1
Exposure to direct cyberbullying (vicious behavior, anonymous bullying, public humiliation, name-calling)	102	6.00	29	77	100
Exposure to indirect cyberbullying (becoming excluded from digital communities, online gossip)	74	4.35	12	59	82
Exposure to sexual harassment and molestation	69	4.06	29	53	82
Exposure to pressures regarding appearance; an appearance-oriented world view	44	2.59	6	35	59
Exposure to negative behavior/provocative material shared by others (e.g., images or video footage of violence, at-risk situations, intoxicants, or gambling)	44	2.59	0	41	65
Exposure to racism	36	2.12	0	35	35
Lack of knowledge and skills to critically address social media content	36	2.12	12	29	53
Reduced quality/quantity of sleep through the use of social media	33	1.94	0	35	47
Addiction to social media use (i.e., compulsive and uncontrolled use)	32	1.88	6	35	41
The child or adolescent behaves offensively on social media and does not understand the emotional content of messages (low emotional skills)	32	1.88	0	24	47
Incapacity to manage time spent on social media	25	1.47	0	18	47
The need to be constantly available in order not to be excluded (fear of missing out)	22	1.29	6	12	35
Sharing of one’s own personal, private, and sensitive information or files	21	1.24	0	12	35
Exposure to online scams	16	0.94	0	12	24
Excessive time spent on social media and increased screen time	14	0.82	0	12	18
Sharing without permission the private and sensitive information or files of other people	12	0.71	0	12	30

Agreement % = 8 is the proportion of respondents who gave the item score a rating of 8. Agreement % ≥ 4 is the proportion of respondents who rated the item score among the top half of items. Agreement % ≥ 1 is the proportion of respondents who rated the item score among the top 8 items.

**Table 4:** Competencies adolescents require to prevent/deal with problematic social media situations; ranked in order of importance by an expert panel.

Competency	Sum	Mean	Agreement % = 8	Agreement % ≥ 4	Agreement % ≥ 1
Ability to act responsibly and without offending others on social media	80	4.71	18	71	77
Knowing what kinds of activity are prohibited (identity theft, sexual harassment, dissemination of information, defamation)	72	4.24	24	65	71
Knowing whom to contact when exposed to cyberbullying, harassment, or sexual harassment	58	3.41	6	47	77
Ability to ask for help from a trusted adult if necessary	46	2.71	12	41	53
Having knowledge and skills on how to apply security practices to protect privacy (one’s own and that others)	40	2.35	6	35	47
Ability to act empathetically and with respect for others on social media	35	2.06	6	18	65
Ability to evaluate the credibility of social media posts; knowing that information given on social media is not the whole truth about the poster’s life	35	2.06	6	18	59
Ability to protect personal privacy (e.g., passwords and profile privacy settings)	33	1.94	0	35	35
Ability to assess the trustworthiness of a previously unknown online friend	32	1.88	6	24	53
Ability to manage time spent on social media	27	1.59	6	18	47
Ability to assess the trustworthiness of published information	26	1.53	0	24	35
Knowing one’s own rights (e.g., right to information, privacy, and freedom of expression)	26	1.53	0	24	29
Ability to assess what contents are suitable for publication or sharing	24	1.41	0	24	35
Ability to assess one's own behavior and that of others on social media	17	1.00	6	12	18
Ability to explain how social media affect one’s self-image and self-esteem	16	0.94	6	12	18
Ability to identify problematic social media situations in one’s daily life	16	0.94	0	12	29
Ability to identify, process, express, and regulate emotions on social media	15	0.88	0	12	24
Ability to compare information published in different data sources	13	0.76	0	12	24
Knowing where to report inappropriate material	1	0.06	0	0	6

Agreement % = 8 is the proportion of respondents who gave the item score a rating of 8. Agreement % ≥ 4 is the proportion of respondents who rated the item score among the top half of the items. Agreement % ≥ 1 is the proportion of respondents who rated the item score among the top 8 items.

In order to identify the most important problematic situations and competencies, sum scores were calculated. As regards the problematic situations, *exposure to direct cyberbullying* received a sum score of 102, while *exposure to indirect cyberbullying* received a sum score of 74. As regards the most important competencies, *the ability to act responsibly and without offending others* received a sum score of 80, while *knowing what kinds of activity are prohibited* received a sum score of 72.

## Discussion

The study investigated experts’ opinions on (1) the most important problematic situations that adolescents may encounter when they use the social media, and (2) the competencies needed by adolescents in addressing these situations. According to the findings, the three most important problematic situations were *exposure to direct cyberbullying* (i.e., vicious behavior, public humiliation), *exposure to indirect cyberbullying* (i.e., being excluded from digital communities), and *exposure to sexual harassment and molestation*. The three most important competencies were *the ability to act responsibly in social media*, *knowing what kinds of activity are prohibited* (e.g., identity theft, dissemination of false information, defamation), and *knowing whom to contact when exposed to cyberbullying, harassment, or sexual harassment*. Despite some differences, the competencies showed a good match with the problematic situations. In addition, some of the competencies identified could be seen as transversal competencies, relevant to many problematic situations (e.g., *the ability to assess one's own behavior and that of others on social media*, *the ability to identify problematic social media situations in one’s daily life*, and *knowing one’s own rights*).

Our findings are in line with previous studies investigating perspectives by experts^10,11^ and adolescents^2,35^ regarding problematic situations in the social media. These have indicated cyberbullying and sexual harassment as particularly problematic. This may be due to their direct negative consequences on the victim's wellbeing, but further research is needed on this aspect. It is worth noting that (direct and indirect) cyberbullying may last longer than bullying in traditional environments (e.g., in schools), due to a lack of immediate indications of bullying, and to the adolescent not mentioning bullying to an adult^54^ Furthermore, Slonje et al.^55^ have noted that cyberbullying can be anonymous, but that the potential audience can be larger; also, that cyberbullying is not tied to any time or place and may take place in usually safe environments (e.g., within the home), meaning that there is no “safe haven.”

In cases of bullying or sexual harassment, or other concerning situations such as racism (which was also ranked fairly high by the experts of this study), it is important that adolescents should not face these experiences on their own. According to the EU Kids Online study^6^, almost half of adolescents had either talked to their parents (40%) or to their peers of the same age (50%) after negative online experiences; however, one in five had not talked to anyone. Many abilities are important in terms of being able to contact someone, including knowing (1) *what kinds of activity are prohibited*, or in other ways unacceptable, (2) *whom to contact when exposed to*, for instance, *cyberbullying, harassment, or sexual harassment*, and (3) *where to report inappropriate material*. The first two competencies were ranked as the second and third most important competencies by the experts in our study. However, the proportions related to not receiving help from parents (36%), friends (55%), or a teacher (65%) after being bothered by something on the internet^6^ indicate clear deficiencies in social support. The experts also highlighted the importance of having the skills to *assess the trustworthiness of the previously unknown online friend* and to *assess what contents are suitable for publication or sharing*. These are critical in hindering exposure not just to harassment, but also to other kinds of security risks, such as privacy violations^56^, and are clearly linked to the problematic situation of *sharing one’s own personal, private, and sensitive information or files*. Competencies related to privacy issues can be deemed particularly important in situations where adolescents share private or sensitive information (their own or that of others), or are exposed to online scams^12^. However, based on the EU Kids Online 2020 report, every fifth adolescent has difficulties in changing their privacy settings^6^.

The expert panel rated social media-induced *pressures regarding appearance* as a significant problematic situation. This concern has also been raised by previous literature in which it has been noted that popular social media platforms contain an abundance of appearance-focused content promoting athletic and muscular ideals for males, and thin and curvaceous ideals for females^57^. This may lead to unrealistic standards of beauty and physical appearance, objectifying self-concepts, and impossible body standards among adolescents^10,26^. The visual nature of the social media, combined with quantifiable peer feedback (e.g., likes, comments) and the public exposure entailed, may exacerbate appearance pressure and appearance-focused social comparison in the developmentally sensitive period of adolescence^58^. Thus, experts in this study highlighted the importance of providing adolescents with the machinery to *evaluate the credibility of social media posts,* and to *explain how social media affect one’s self-image and self-esteem*.

Another important problematic situation in the views of the experts was *exposure to negative behavior/provocative material* (e.g., images or video footage of violence, at-risk situations, intoxicants). According to earlier models and theories, including the *Facebook Influence Model*^59^ and the *Super-peer* theory^60^, the social media context amplifies peer influence processes, which may lead to participation in and the publishing of risky behavior, with the hope of measurable validation (e.g., likes). Furthermore, the social media environment could potentially intensify the pursuit of sensation by presenting risky challenges as thrilling and enjoyable^1^. Although among some adolescents provocative material (such as violence) may induce excitement, in others it may cause anxiety, fear, and depressive feelings^61,62^. When exposed to such material, adolescents should be equipped with skills to evaluate the post's credibility, assess the publisher's behavior and reasoning behind the post, and know where to report the inappropriate material—competencies also deemed important by the expert panel.

According to our study, the most important problematic situations relate to adolescents having the role of an object (i.e., being a victim or “being exposed to” various problematic situations) rather than that of a perpetrator (involving, for example, offensive behavior, and the sharing of personal or sensitive files belonging to others), with the perpetrator’s role being ranked at 10th or lower in order of importance. However, the competencies that emerged as high in the ranking covered not just the skills needed to deal with being treated as an object in social media communication (such as the abilities to identify what behavior is not right in the social media, and how to proceed if one is faced with such situations), but also the competencies to avoid such situations in the role of a communicator. The latter would involve the social media competencies covered by, for example, *the ability to act responsibly and without offending others*, *the ability to act empathetically and with respect for others*, and *the ability to assess one's own behavior and that of others.* This clearly underlines the dual role of adolescents in the social media. At the same time, it echoes discussions on “digital citizenship,” going beyond the mere emphasis on how to be safe from digital risks, towards highlighting the role of “the rights and responsibilities of individuals and groups as communicators,” encompassing also online communication^63^. The need in question is also highlighted by the declaration of the Council of Europe^64^, which refers to “the ability to engage positively, critically and competently in the digital environment, drawing on the skills of effective communication and creation, to practice forms of social participation that are respectful of human rights and dignity through the responsible use of technology”.

The socio-emotional skills (such as *the ability to act empathetically and with respect for others on social media*) and self-regulatory competencies (such as *the ability to manage time spent on social media*) that were ranked highly by the experts have been deemed important in previous studies (for socio-emotional skills, see^65^, for self-regulatory competencies, see^34^). Overall, a large body of literature [e.g.,^66,67^] confirms that vicious online behavior (such as cyberbullying) can be explained by a lack of socio-emotional skills during adolescence. Cyber perpetrators have been shown to have low empathy in the affective domain, but also low cognitive empathy^66^. On the other hand, low social and emotional efficiency has also been linked to an increased likelihood of becoming a cybervictim^65^. It further appears to be the case that weak self-regulatory competencies among adolescents may lead to problematic use of social media^68^, intensive use^69^, and nighttime-specific use^70^. These notions are in line with the Delphi findings; several identified problematic situations had a link with either the time spent on the social media (e.g., excessive time spent on social media, and incapacity to manage that time), or how the time spent affected one’s behavior (e.g., sleeping patterns, addictive use of social media, and the need to be constantly available to avoid exclusion). In Europe and Canada, almost every tenth adolescent can be seen as a problematic social media user^71^. Furthermore, the proportion of those with a heightened risk of developing such a behavioral pattern is even bigger. For instance in Finland, every third adolescent can be seen as belonging to a group with a heightened risk for problematic social media use^68^. Given the well-established literature showing the link between problematic use and unfavorable health and health behavior [e.g.,^68–70^], it is imperative that adolescents are provided with learning experiences that could improve their self-regulative competencies in the relevant online contexts^72^.

Despite media interest in Finland during the last couple of years, the scores of the experts were too low to move *exposure to social media challenges that are harmful or dangerous to health* or *exposure to distorted or false information* (e.g., fake news, conspiracy theories) to the third Delphi round. Research is needed to understand these findings, given the possible severe consequences of risky behavior (as in being severely burned after a climb to the roof of a train). Moreover, mis- and disinformation is a problem that almost all people face in the social media, with possible danger to health. However, one particular problematic situation, namely *a lack of knowledge and skills to critically address social media content*, and several skills such as *the ability* to *assess the trustworthiness of published information*, and *the ability to compare information published in different data sources*, raised by the experts in this study, echo similar problems (plus the skills to handle them) raised elsewhere. In Europe, while 60% of adolescents report being able to assess the validity of online information, 40% do not^6^. However, recent PISA findings indicate an even worse situation, insofar as only 7% of the students were able to find the differences “between fact and opinion as applied to complex or abstract statements”^73^. Much work is needed to further the aim that “no child should be left behind in the digital age, especially not those already disadvantaged in other ways”^6^.

The strengths of the study included a versatile profile of experts, identified via the guidelines of Okoli and Pawlowski^46^. Furthermore, the anonymity of the responses reduced the impact of dominant individuals and peer pressure to conform, thus allowing opinions to be considered in a non-adversarial manner^46^. In a Delphi study, the responses are weighted equally, so individual answers cannot shift the opinions of the group. Despite this, the current study has limitations which could open avenues for future research. For example, the study could be viewed as limited by the lack of clear methodological guidelines for the Delphi design. Furthermore, the arbitrary cut-off in the second round was due to there being no generally accepted criteria in the literature^53^. Note also that the time and place of participation were not controlled. The study was further limited by cultural and geographical factors, since it only involved Finnish experts. One can surmise that in other countries, there could be differences in expert views regarding the most important problematic situations encountered on the social media, as well as the competencies required to deal with them. It is therefore important to be cautious in generalizing the findings beyond Finland. One should also bear in mind that the experts’ views were subjective; thus, it is possible that another Delphi panel with the same questions would come to different conclusions. Furthermore, the first round of the Delphi study carried the risk of biased interpretation, even if this was considered by the research team by carefully going through the expert answers, and serving as critical friends for each other^49^.

Future studies could (1) test the effectiveness of interventions aimed at applying the identified skills to problematic situations, and (2) study adolescents’ own views on the problematic situations and relevant competencies, with possibilities for contrasting these with the views of experts. Note also that different platforms may be differently associated with problematic situations; hence, a platform-specific approach would be beneficial, in parallel with differentiating between social media activities in general. However, as suggested by Smahel et al.^6^, the degree to which children are exposed to online risks is often less than that feared by parents or claimed by the mass media. There is a need for a deeper understanding of which adolescents are most susceptible to online risks. Studies on these lines would be of great value in developing intervention programs, educational settings, and policies applicable to wellbeing in the digital world.

To conclude, online spaces, including the social media, form important contexts for the growth and development of adolescents, and increased time spent on social media has been linked to a higher likelihood of problematic situations^1^. Hence, developing the competencies to address such situations becomes crucial. This study can be viewed as identifying the most important problematic situations and related competencies, meaning that the results could be applied to intervention programs, the educational settings of professionals (such as teachers and social workers), the information given to parents, and political decision-making.

## Data Availability

The datasets used and/or analyzed during the current study are available from the corresponding author on reasonable request. The privacy notice compliant with the European Union's General Data Protection Regulation (GDPR)^51^, approved by the participants via informed consent will be considered in data sharing.
